# *In Vitro* Synergy of Biochanin A and Ciprofloxacin against Clinical Isolates of *Staphylococcus aureus*

**DOI:** 10.3390/molecules16086656

**Published:** 2011-08-05

**Authors:** Guoxing Liu, Jun-Chao Liang, Xue-Lin Wang, Zhao-Hui Li, Wei Wang, Na Guo, Xiu-Ping Wu, Feng-Ge Shen, Ming-Xun Xing, Li-Hui Liu, Lei Li, Ming-Yuan Liu, Lu Yu

**Affiliations:** 1 Key Laboratory of Zoonosis Research, Ministry of Education, Institute of Zoonosis, College of Animal Science and Veterinary Medicine, Jilin University, Changchun 130062, China; 2 Department of Food Quality and Safety, College of Quartermaster Technology, Jilin University, Changchun 130062, China; 3 Institute of Pathogen Biology, Chinese Academy of Medical Sciences, Beijing 100730, China; 4 State Key Laboratory for Molecular Virology and Genetic Engineering, Chinese Center for Disease Control and Prevention, Beijing 100176, China

**Keywords:** antimycobacterial, biochanin A, ciprofloxacin, *Staphylococcus aureus*, synergy

## Abstract

Many clinical isolates of *Staphylococcus aureus* (*S. aureus*) are resistant to numerous antimicrobials, including the fluoroquinolones (FQs). Flavonoids such as biochanin A (BCA) are compounds that are naturally present in fruits, vegetables, and plant-derived beverages. The goal of this investigation was to study the possible synergy between the antimicrobial agents BCA and ciprofloxacin (CPFX) when used in combination; CPFX was chosen as a representative FQ compound. We used *S. aureus* strain ATCC 25923 and 11 fluoroquinolone (FQ)-resistant methicillin-resistant *S. aureus* (MRSA) strains. Results from the drug susceptibility testing and checkerboard assays show that the minimum inhibitory concentration (MIC) of BCA ranged from 64 µg/mL to 512 µg/mL. When BCA was combined with CPFX, the fractional inhibitory concentration index (FICI) data showed that there was synergy in all 12 of the *S. aureus* strains tested. No antagonistic activity was observed in any of the strains tested. The results of time-kill tests and agar diffusion tests confirm that there was synergy between BCA and CPFX against *S. aureus* strains. These results suggest that BCA can be combined with FQs to produce a powerful antimicrobial agent.

## 1. Introduction

*Staphylococcus aureus* (*S*. *aureus*) is a significant community and nosocomially acquired pathogen that colonizes the nasal passages and skin of healthy people [1]. *S. aureus* colonization can lead to local and systemic infections in humans, which can range from superficial skin infections to life-threatening pneumonia or sepsis [2]. The widespread use of methicillin and other semi-synthetic penicillins in the late 1960s led to the emergence of the strains of methicillin resistant *S*. *aureus* (MRSA) [3]. Current more than 60% of *S. aureus* isolates are resistant to methicillin, and a number of strains have developed resistance to more than 20 other antimicrobial drugs [4], including the fluoroquinolones (FQs). Because of this rapidly evolving drug resistance, the morbidity and mortality associated with *S. aureus* infections is high in spite of antimicrobial therapy [5]. A novel approach for treating resistant strains of bacteria is to use a combination therapy, which may improve the efficacy of antimicrobial treatment for resistant microbes.

**Figure 1:**
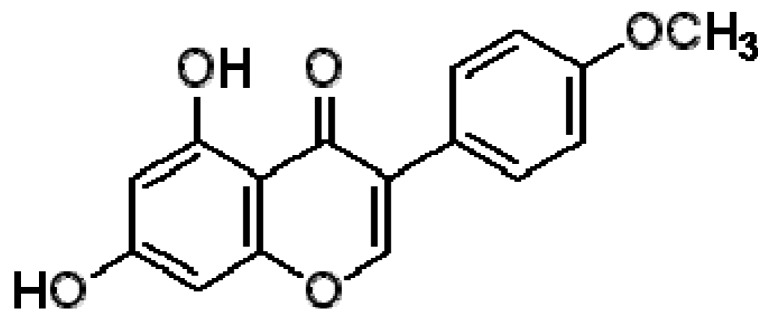
Chemical formula of Biochanin A.

Antimicrobials can have detrimental side effects, and as mentioned previously, their overuse can lead to bacterial resistance. Thus, plant-derived products have been explored for use as alternative treatments [6,7]. *In vitro* studies have shown that several flavonoids have a strong antimicrobial activity against gram-positive bacteria [6,8]. Biochanin A (BCA, Figure 1), an isoflavonoid found in chickpea (*Cicer arietinum*), in peanuts and in other legumes, inhibits the growth of various microorganisms [9,10,11,12]. BCA is classified as a phytoestrogen and has putative benefits in dietary cancer prophylaxis [13]. It acts as a chemopreventive agent by inhibiting the metabolism of benzo(a)pyren, which is the initial step in its carcinogenicity [14,15,16]. In addition, BCA was found to be potent agonists of aryl hydrocarbon receptor *in vitro*, which activation affects cell cycle control and apoptosis [13]. As a common plant product, BCA is generally considered to be nontoxic [12]. Our preliminary data show that BCA has antimicrobial activity against several *S. aureus* strains. These interesting results motivated us to further study possible synergy between BCA and a representative FQ (CPFX) against several drug resistant *S. aureus* isolates. In this study, we investigated the combined effects of BCA and CPFX against clinical isolates of *S. aureus* with the checkerboard microdilution method, time-killing tests, and agar diffusion tests.

## 2. Results

### 2.1. Drug Effect Alone and in Combination

In the assessment of the antimicrobial activities of the drug alone, the MIC values for BCA treatment alone against the 11 clinical isolates ranged from 128 to 512 µg/mL; the MIC values for CPFX treatment alone against the 11 clinical isolate strains ranged from 128 to 256 µg/mL (Table 1), and these large values show that the clinical MRSA isolates are resistant to CPFX and possibly a majority of the FQs. The MIC values of BCA and CPFX against ATCC 25923 strain were 64 and 1 µg/mL, respectively, and the CPFX MIC value is within the reference range. These results show that BCA by itself has weak antimicrobial activity against the drug-resistant clinical *S. aureus* isolates.

In the combination study, after the background OD_660_ obtained from the microorganism-free well had been subtracted, the percentages of bacterial growth for the combination were calculated by comparing the OD_660_ of the drug-containing well with that of the drug-free well. In the checkerboard assay, FICI was calculated to analyze the interaction between the two drugs (BCA and CPFX) when combined. Of the 12 total *S. aureus* strains that were tested, the interaction between BCA and CPFX was synergistic in all of the strains according to the FICI method (all of the FICI values ranged from 0.06 to 0.5) (Table 2). We did not find any antagonistic interaction between BCA and CPFX.

From the data above, we conclude that when used alone, BCA has very weak antimicrobial activity. However, BCA in combination with CPFX shows potent antimicrobial effects against either FQ-resistant MRSA isolates or sensitive strains. These results were verified by the following two independent methods: Agar diffusion and time-kill curves.

### 2.2. Time-Kill Curves

**Figure 2:**
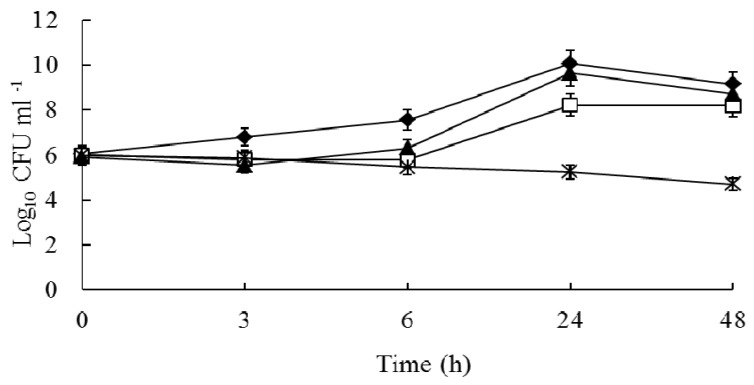
Time-kill curves of BCA and CPFX alone and in combination against clinical Methicillin -resistant *S. aureus* isolate 1662. The strain at starting inocula of 10^6^ CFU/mL were exposed to *in vivo*-achievable concentrations of 32 µg/mL BCA alone and in combination with 64 µg/mL CPFX. At 0, 3, 6, 24, and 48 h, aliquots were removed from each test tube to examine the cell metabolism ability. Symbols: 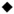, no drug control; □, CPFX; ▲, BCA; *, CPFX plus BCA.

Synergy with the drug combination was also seen in the time-kill curves (Figure 2). The time-kill studies were conducted using BCA and CPFX alone and in combination against one chosen clinical FQ-resistant MRSA 1662 isolate. After 24 h, BCA (32 µg/mL) or CPFX (32 µg/mL) alone did not significantly affect the growth curve. The combination of CPFX and BCA had a higher antimicrobial effect than either drug alone. Given the initial inoculum of 10^6^ CFU/mL, CPFX in combination with BCA resulted in a 2.6-log_10_ CFU/mL decrease compared to 32 µg/mL CPFX alone at 24 h for isolate FQ-resistant MRSA 1662.

### 2.3. Agar Diffusion Test

**Figure 3:**
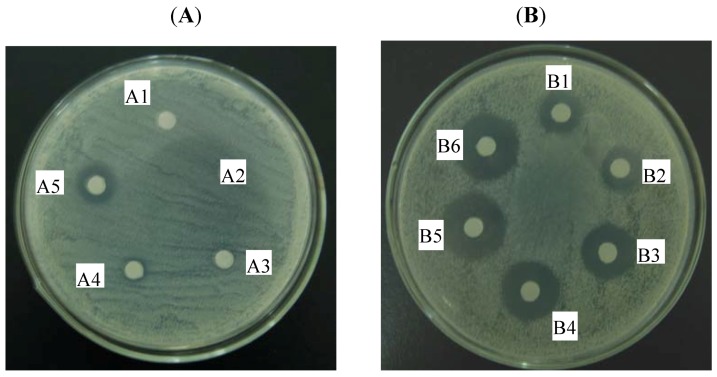
Agar disk diffusion assay with various concentrations of BCA alone (**A**) or in combination with CPFX (**B**) against *S. aureus* isolate MRSA 1662. Lables: A1, CONTROL; A2, BCA 32 µg/mL; A3, BCA 64 µg/mL; A4, BCA 128 µg/mL; A5, BCA 256 µg/mL; B1, CPFX 128 µg/mL; B2, CPFX 128 µg/mL + BCA 16 µg/mL; B3, CPFX 128 µg/mL + BCA 32 µg/mL; B4, CPFX 128 µg/mL + BCA 64 µg/mL; B5, CPFX 128 µg/mL + BCA 128 µg/mL; B6, CPFX 128 µg/mL + BCA 256 µg/mL.

The agar diffusion test was used to visually detect synergy between BCA and CPFX when combined (Figure 3). BCA alone (64 µg) had no antimicrobial activity against the FQ-resistant MRSA 1662 isolate, while CPFX (128 µg) showed weak antimicrobial activity. However, BCA (128 µg and 256 µg) in combination with CPFX (128 µg) showed better antimicrobial effects. The combination of BCA and CPFX resulted in clearer and larger zones compared to either drug alone. The inhibition zone radius was increased to 10.0, 13.5, 14.0, 15.5 and 16.0 mm around the disks that contained CPFX (128 µg) plus various amounts of BCA (16, 32, 64, 128, and 256 µg, respectively). These results were similar to the results of the checkerboard microdilution assay.

## 3. Discussion

In this study, we used the checkerboard microdilution method to analyze the effects of CPFX and BCA combinations on *S. aureus*. Subsequently, the time-kill and agar diffusion assays were used to further examine the nature of these interactions and to further analyze the interaction between BCA and CPFX against isolate FQ-resistant MRSA 1662 isolate.

We used the FICI to interpret our checkerboard method results, although interpretation of synergy or antagonism between drugs with the FICI method can be problematic. Because MIC determinations ares sensitive to dilution errors, the MIC is usually taken to be accurate to within one dilution when two-fold serial dilutions are used. Because the FICIs are determined from two drugs, the FICI value may differ between experiments. Therefore, in the interpretation of a single FICI, a value of >4 is considered to indicate antagonism, while a value of ≤0.5 is considered to indicate synergy [17].

Previous studies have shown that when experiments were performed in triplicate for each strain combination studied, the results of the FICI model for all replicates could be interpreted as a single outcome (synergy, antagonism or indifference), thereby constraining the inter-experimental error. Thus, when the results of all three replicates were concordant, synergy or antagonism was claimed if FICIs were below 1 or above 1, respectively [18]. In the study, all cases show synergism.

The time-kill tubes were vortexed, a sample was removed at various time points, and the colony count was determined. Figure 2 clearly showed that the growth of FQ-resistant MRSA 1662 isolate exposed to the combination of CPFX and BCA was significantly inhibited compaierd to either drug alone, but the cells present in the inoculum were still viable upon transfer. This meant the effect was bacteriostatic, which is consistent with ciprofloxacin’s mechanism of action. A bactericidal effect was defined as a ≥3-log10 CFU/mL decrease after 24 h of incubation compared to the size of the initial inoculum. Synergism was defined as a decrease in the colony count of ≥2-log10 CFU/mL with the combination compared to the count obtained with the most active single drug [31]. As the results showed, there was a 2.6-log_10_ CFU/mL decrease with CPFX in combination with BCA compared to 32 µg/mL CPFX alone at 24 h for isolate FQ-resistant MRSA 1662, which meant CPFX and BCA had synergetic bacteriostatic effect. For these experiments, the FICI analysis agreed well with the time-kill curves for the strains that were tested. The agar diffusion method can provide visual results of the interaction between BCA and CPFX. We found that the results of the agar diffusion, checkerboard microdilution and the time-kill experiments were in good agreement.

BCA is a selective drug that has strong antifungal activity [19,20,21] and can suppress the growth of various microorganisms [22]. The data from this study show that BCA alone has moderate MIC values for the methicillin-susceptible *S. aureus* strains. However, BCA can potentiate the antimicrobial activity of representative FQs (ciprofloxacin) against methicillin-resistant *S. aureus* strains, besides its direct inhibitory effect on microorganisms. The BCA/CPFX combination produced a powerful drug that was more effective than either drug alone. Flavonoids can complex with extracellular and soluble proteins and can thus complex with bacterial cell walls and disrupt microbial membranes [8,23]. Genistein is a topoisomerase inhibitor that has a structure similar to BCA (except for one surplus methyl group at the 4’ position); thus, BCA may also inhibit DNA topoisomerases, which participate in various aspects of DNA metabolism to inhibit microorganisms, the interference related metabolic pathways may be responsible for the antimicrobial action of BCA [12]. A study by Lee showed that a methoxyl group and a hydroxyl group at the 5’ position were essential for antimicrobial activity [12], and both of these criteria are present in the BCA structure (Figure 1).

FQs are some of the most frequently prescribed antimicrobial agents worldwide. FQs interact with DNA gyrase, bacterial type II topoisomerase gyrase, and topoisomerase IV, which leads to changes in DNA supercoiling and ultimately affects gene transcription and DNA metabolism [24,25,26]. Previous study showed FQs can induce the SOS response by comparison of a wild-type *S. aureus* strain, which is a critical response to environmental stress [27]. Thus, FQs increase the longevity of the normally short-lived DNA-topoisomerase cleavage intermediates [28]. These intermediates interfere with the DNA machinery, which results in multiple deleterious effects, such as chromosome fragmentation, inhibition of DNA synthesis, and death [29]. Thus, BCA and CPFX have their own antimicrobial mechanism, it is necessary to determine the underlying mechanism of BCA/CPFX synergy in our further study.

## 4. Experimental

### 4.1. Bacterial Strains and Reagents

Eleven clinical MRSA isolates were obtained from blood samples of infected patients who were admitted to the First Hospital of Jilin University during the year 2008–2009. The quality control (QC) strain ATCC 25923 was obtained from the China Medical Culture Collection Center (CMCC). BCA, dimethyl sulfoxide (DMSO) and CPFX were purchased from Sigma (St. Louis, MO, USA).

### 4.2. Medium

Mueller-Hinton broth (MHB) and agar (MHA) were purchased from BD Biosciences, Inc. (Sparks, MD, USA). The *S. aureus* colony counts were determined from growth on the MHA.

### 4.3. Drug Susceptibility Testing

The MICs of drugs were determined by microdilution of the drug in MHB, which was performed as recommended by the Clinical and Laboratory Standards Institute (CLSI, formerly NCCLS [30]). The strains were plated on MHA and incubated at 37 °C overnight. Colonies from the plates were resuspended in MHB, and the concentration was adjusted to an OD_660_ of 0.1 (~10^8^ CFU/mL, which matches a 0.5 McFarland turbidity standard). The inoculum was diluted in MHB to a concentration of 10^6^ CFU/mL. Serial two-fold dilutions of the antimicrobial agents were prepared in MHB to obtain the required range of concentrations. The assay was performed in 96-well microtiter plates, and a positive control, which contained inoculated broth without drugs, was included in every plate. Fifty microliters of the diluted antimicrobial drug was plated into individual wells in triplicate. Fifty microliters of the bacterial inoculum was added to each well, and the plate was incubated at 37 °C for 24 h. Bacterial growth was examined by measuring the optical density at 660 nm with a microplate spectrophotometer. The MIC was defined as the lowest concentration of drug that inhibited >90% of the microorganism’s growth [17]. Each isolate was tested in triplicate on different days.

### 4.4. Checkerboard Assay

Interactions between CPFX and BCA were assessed with a microbroth checkerboard technique. Serial two-fold dilutions of BCA and CPFX were prepared in MHB that ranged from 1/512 to 4-fold MIC concentration. The checkerboard plates were inoculated with 10^6^ CFU/mL of MRSA 1662 and incubated for 24 h at 37 °C. The growth in each well was quantified spectrophotometrically as previously described above, and the background OD_660_ was subtracted from the OD_660_ of each well. The percentage of growth in each well was calculated by dividing the OD_660_ of each well by the OD_660_ of the drug-free well, and the plates were handled according to the protocol previously published by Li *et al.* [18]. Each isolate was tested in triplicate on different days.

### 4.5. Drug Interaction Interpretation

To study the interaction of BCA in combination with CPFX against *S. aureus*, the data were analyzed with the models: The fractional inhibitory concentration index (FICI).

The FICI was calculated as follows: FICI = FIC_A_ + FIC_B_, where FIC_A_ = MIC of drug A in the combination divided by the MIC of drug A alone, and FIC_B_ = MIC of drug B in the combination divided by the MIC of drug B alone. Synergy was defined when the FICI was ≤0.5, indifference when the FICI fell in the range of 0.5–4, and antagonism was defined as an FICI >4 [17].

### 4.6. Time-Kill Curves

The tested strain MRSA 1662 was prepared at a concentration of 10^6^ CFU/mL in MHB [31]. Tubes of broth were prepared containing CPFX alone at a concentration of 1/8 the MIC, BCA alone at a concentration of 1/8 the MIC, and a combination of both drugs (1/8 the MIC of CPFX and 1/8 the MIC of BCA). A control tube without drugs was included in each series. At predetermined time points (0, 3, 6, 24, and 48 h), a 100-μL sample was removed from each test suspension, serially diluted in MHB, and plated on MHA plates to determine the colony count [32]. The experiment was performed triplicately.

A bactericidal effect was defined as a ≥3-log10 CFU/mL decrease after 24 h of incubation compared to the size of the initial inoculum. Synergism was defined as a decrease in the colony count of ≥2-log10 CFU/mL with the combination compared to the count obtained with the most active single drug. Antagonism was defined as an increase in the colony count of ≥2-log10 CFU/mL with the combination compared to the count obtained with the most active single drug [31].

### 4.7. Agar Diffusion Test

The interaction between BCA and CPFX was tested with an agar diffusion assay. Disks containing BCA and CPFX alone and in combination were placed on the MHA plates spread with MRSA 1662 [33]. After incubation for 24 h at 37 °C, the zone of inhibition was measured.

## 5. Conclusions

In summary, this is first report that BCA displays synergistic activity in combination with the antibacterial agent CPFX against *S. aureus*, which might provide an alternative approach to overcome antimicrobial drug resistance.

## Figures and Tables

**Table 1:** Susceptibilities of BCA alone and in combination with CPFX against *S. aureus* strains, as obtained by the spectrophotometric method.

Strains	Median MIC (range) of drug (µg/mL)			
	Alone		In combination	
	BCA	CPFX	BCA	CPFX
*S. aureus* 1662	256 (128–256)	256 (256)	32 (32–64)	32 (32–64)
*S. aureus* 3633	128 (128–256)	256 (256)	16 (8–16)	8 (8)
*S. aureus* 3814	128 (128)	256 (128–256)	32 (16–32)	64 (32–64)
*S. aureus* 3725	256 (256–512)	256 (256)	32 (32–64)	16 (16–32)
*S. aureus* 2855	128 (128–256)	256 (128–256)	8 (8–16)	16 (8–16)
*S. aureus* 1881	128 (128–256)	256 (256)	32 (32–64)	32 (32–64)
*S. aureus* 3014	256 (256)	256 (128–256)	32 (32–64)	32 (32–64)
*S. aureus* 3115	256 (128–256)	128 (128–256)	64 (32–64)	32 (32–64)
*S. aureus* 3451	256 (256–512)	256 (256–512)	32 (32–64)	8 (8–16)
*S. aureus* 3182	256 (256–512)	256 (256)	64 (64–128)	32 (32)
*S. aureus* 1787	512 (256–512)	256 (128–256)	64 (32–64)	32 (32–64)
*S.aureus* ATCC 25923	64 (64)	1 (1)	16 (4–16)	0.25 (0.25)

**Table 2:** *In vitro* interaction between CPFX and BCA.

Strains	Nonparametric method *	
	Fractional Inhibitory Concentration Index (FICI)	
	Mean(range)	Interpretation
*S. aureus* 1662	0.25 (0.25)	SYN
*S. aureus* 3633	0.16 (0.06–0.16)	SYN
*S. aureus* 3814	0.38 (0.25–0.50)	SYN
*S. aureus* 3725	0.19 (0.09–0.31)	SYN
*S. aureus* 2855	0.13 (0.06–0.25)	SYN
*S. aureus* 1881	0.38 (0.38–0.50)	SYN
*S. aureus* 3014	0.38 (0.25–0.5)	SYN
*S. aureus* 3115	0.50 (0.38–0.50)	SYN
*S. aureus* 3451	0.16 (0.08–0.31)	SYN
*S. aureus* 3182	0.38 (0.38)	SYN
*S. aureus* 1787	0.25 (0.25–0.38)	SYN
*S.aureus* ATCC 25923	0.50 (0.31–0.50)	SYN

* SYN, synergism. For the FICI model, synergy was defined as a FICI of ≤0.5, antagonism was defined as a FICI of >4.0, and indifference was defined as a FICI of >0.5 to 4 (*i.e.*, no interaction).
